# Automatically determining cause of death from verbal autopsy narratives

**DOI:** 10.1186/s12911-019-0841-9

**Published:** 2019-07-09

**Authors:** Serena Jeblee, Mireille Gomes, Prabhat Jha, Frank Rudzicz, Graeme Hirst

**Affiliations:** 1 Department of Computer Science, University of Toronto, Toronto, Canada; 2 Vector Institute for Artificial Intelligence, Toronto, Canada; 3 Centre for Global Health Research, St.Michael’s Hospital, Toronto, Canada; 4 Dalla Lana School of Public Health, University of Toronto, Toronto, Canada; 5 Li Ka Shing Knowledge Institute, St Michael’s Hospital, Toronto, Canada; 6 Surgical Safety Technologies Inc, Toronto, Canada

**Keywords:** Cause of death, Computer-coded verbal autopsy (CCVA), Physician-certified verbal autopsy (PCVA), Machine learning, Natural language processing, Tariff method, Verbal autopsy

## Abstract

**Background:**

A verbal autopsy (VA) is a post-hoc written interview report of the symptoms preceding a person’s death in cases where no official cause of death (CoD) was determined by a physician. Current leading automated VA coding methods primarily use structured data from VAs to assign a CoD category. We present a method to automatically determine CoD categories from VA free-text narratives alone.

**Methods:**

After preprocessing and spelling correction, our method extracts word frequency counts from the narratives and uses them as input to four different machine learning classifiers: naïve Bayes, random forest, support vector machines, and a neural network.

**Results:**

For individual CoD classification, our best classifier achieves a sensitivity of.770 for adult deaths for 15 CoD categories (as compared to the current best reported sensitivity of.57), and.662 with 48 WHO categories. When predicting the CoD distribution at the population level, our best classifier achieves.962 cause-specific mortality fraction accuracy for 15 categories and.908 for 48 categories, which is on par with leading CoD distribution estimation methods.

**Conclusions:**

Our narrative-based machine learning classifier performs as well as classifiers based on structured data at the individual level. Moreover, our method demonstrates that VA narratives provide important information that can be used by a machine learning system for automated CoD classification. Unlike the structured questionnaire-based methods, this method can be applied to any verbal autopsy dataset, regardless of the collection process or country of origin.

**Electronic supplementary material:**

The online version of this article (10.1186/s12911-019-0841-9) contains supplementary material, which is available to authorized users.

## Background

### Verbal autopsies

Two-thirds of the world’s 60 million deaths each year do not have a known cause of death (CoD). The largest gap between known and unknown CoDs is in developing countries, where many deaths occur at home rather than in health facilities [1]. Verbal autopsy (VA) surveys can help to bridge this gap by providing information about the most prevalent causes, which helps to inform public health planning and resource allocation [2]. A VA survey typically involves interviews with family members of the deceased, conducted by non-medical staff who complete a structured questionnaire about the person’s symptoms and risk factors before death. They also ask the family members about the events and circumstances around the time of death and record the responses in a free-text narrative. Typically, two or more physicians review each completed VA survey and independently make a CoD diagnosis [3], with reconciliation done by another more senior physician if necessary.

Although there have been criticisms of physician-coded VAs [4], there is no other gold standard for VA coding that we can evaluate against, since for most VAs we have no way of knowing the true CoD. Records of hospital deaths cannot be considered a gold standard for non-hospital deaths because of the differences in the distribution of CoDs, as well as the differences between the characteristics of patients who receive care in hospitals and those who die at home without medical attention (such as education level, access to hospital care, types of pathogens, etc.) [3, 5, 6]. For this reason, physician-coded VAs are often used for training and testing automated CoD coding methods.

Automated CoD coding may help to reduce physician time and costs when coding VA surveys. For example, an automated system could be used as a first pass for coding new VA records, where the results could be reviewed (and corrected if necessary) by one physician. This process would still allow for human verification, but would reduce the time and number of physicians needed to look at each record. Since these models can also produce a confidence score for each code, low-confidence output could be flagged for human review.

So far, machine learning techniques have been primarily applied to data from the structured questionnaires only, with the best sensitivity scores around.60 for individual CoD classification, using various numbers of CoD categories (typically 15–30) [7]. Some studies have suggested that the narrative section is unnecessary or of limited use for determining CoD [8]. However, we hypothesize that using the structured questions alone results in insufficient accuracy because information that appears only in the free-text narrative is often essential to making a correct diagnosis, such as symptom chronology and treatment history [9]. Our method uses word frequency counts from the narrative to determine the appropriate CoD category for a VA record. We explore several different models including naïve Bayes, random forests, support vector machines, and a neural network.

### Metrics

In the absence of medical death certification in low- and middle-income countries, VAs are primarily used to estimate the proportion of deaths from various causes at the population level, so as to inform public health planning. Subsequently, individual level VA CoD assignments are often aggregated to determine the CoD distribution in the population.

To evaluate CoD classification at the individual level, we report precision (positive predictive value), sensitivity (recall), and F _1_-measure (the harmonic mean of precision and sensitivity), as well as partial chance-corrected concordance (PCCC). Chance-corrected concordance (CCC) is a measure of how well the predicted CoD categories correspond to the correct CoD categories, and PCCC is the same measure adjusted for the number of possible categories [10]. To evaluate the CoD distribution prediction at the population level, we report Cause-Specific Mortality Fraction (CSMF) accuracy [10, 11]. CSMFs measure the relative proportions of CoDs in a population, and CSMF accuracy measures the similarity of the distribution of CoD categories assigned by the classifier to the true distribution.

However, CSMF accuracy scores of.50 or above can often be achieved by random guessing, especially if the method takes into account the training distribution. So we also report chance-corrected CSMF accuracy (CCCSMFA) [12], which produces a score of 0 for chance performance, and a negative score for performance worse than chance.

### Previous work

Several expert-driven and machine learning methods have been used for automatically categorizing VAs by CoD, at both the individual and the population level [13–19]. Many of these methods are based on questionnaires such as the World Health Organization (WHO) 2016 Verbal Autopsy Instrument [20], which is a standardized VA questionnaire with detailed questions about the subject’s symptoms and medical history.

Boulle et al. [13] were among the first to use neural networks for VA CoD classification in 2001. They used a small set of structured questionnaire data with a neural network and achieved a sensitivity of.453 for individual classification into 16 CoD categories. However, to our knowledge, no current VA coding method uses neural networks despite their recent popularity.

The King-Lu method [21] uses the conditional probability distributions of symptoms to estimate the CoD distribution of a dataset over 13 categories. It does not provide a CoD for individual records. Desai et al. [7] reported a CSMF accuracy of.96 using the King-Lu method on the Indian Million Death Study dataset [3].

InterVA-4, a popular automated VA coding method developed by Byass et al. [14], uses a predetermined list of symptoms and risk factors extracted from a structured questionnaire. Records are assigned one of 62 CoD categories from the WHO 2012 VA Instrument [22] based on conditional probabilities for each symptom given a CoD, as assigned by medical experts, as well as the probabilities of the CoDs themselves. Miasnikof et al. [17] reported a sensitivity of.43 and CSMF accuracy of.71 for InterVA-4 on data from the Million Death Study [3].

InSilicoVA, described by McCormick et al. [15], is a statistical tool that uses a hierarchical Bayesian framework to estimate the CoD for individual records as well as the population distribution. They reported a mean sensitivity of.341 across 34 CoD categories for individual records, and.85 CSMF accuracy.

The Tariff Method, presented by James et al. [16, 23], uses a sum of weighted scores (tariffs) to determine the most probable CoD. The score for each of the possible CoDs is the weighted sum of different tariffs, which are each calculated from the value of a certain indicator (usually a symptom or risk factor). Most of these indicators are taken from the structured questionnaire, although there are also tariffs that represent the presence of some frequent narrative words (50 or more occurrences in the training data). James et al. reported.505 CCC and.770 CSMF accuracy for adult records from the Population Health Metrics Research Consortium (PHMRC) dataset [24], using 53 CoD categories.

Miasnikof et al. [17] used a naïve Bayes classifier to assign CoD categories. They evaluated their classifier on several different datasets, including the PHMRC dataset and the Million Death Study dataset [3, 25], which we will use in this paper (see “Results” section), with 16 CoD categories. They obtained results that surpassed those of the Tariff Method and InterVA-4, including a sensitivity of.57 and CSMF accuracy of.88. However, their model used only data from the structured questionnaire.

Danso et al. [18] used word frequency counts and tf ·idf scores (the frequency of a term divided by the frequency of documents in which it occurs) from VA narratives as features (measurable characteristics of data that are used as input to computational models) with a support vector machine (SVM) classifier, achieving a maximum F _1_ score of.419. They also used a naïve Bayes classifier and a random forest classifier, which achieved F _1_ scores of.373 and.149 respectively. They did not report population level metrics.

Danso et al. [19] used a variety of linguistic features such as part-of-speech tags, noun phrases, and word pairs from 6407 VA narratives of infant deaths from Ghana, and classified the records into 16 CoD categories, achieving a sensitivity of.406 using only the narrative-based features and.616 using a combination of narrative and structured questionnaire features. They noted that they achieved better performance with the linguistic features than with only word occurrence features, though their dataset was small and the part-of-speech tagger was not trained on medical data, and thus is likely to produce incorrect part-of-speech information.

## Methods

### Data

Our main dataset comes from the Million Death Study (MDS), the goal of which is to provide a national estimate of the leading CoDs in India in order to enable evidence-based health programming [3, 25]. Since the majority of available records in MDS are scans of handwritten forms, which are not usable by our automated prediction tool, we use a subset consisting of the records with narratives that have been transcribed into a digital format. This dataset consists mostly of English narratives, which tend to come from southern and northeastern India. However, all states are represented in this dataset. The remaining narratives have been translated into English from various local languages. In addition to this dataset, we also have a set of records from a recent multi-centre randomized control trial (RCT) that was conducted in four districts within two states of India: Gujarat and Punjab, on 9374 deaths [26]. The aim of this RCT was to assess whether current leading machine learning algorithms perform as well as physician diagnosis when determining the CoD for VAs at the population level. The RCT collected VAs on all deaths from the study sites up to age 70 that occurred within five years preceding the study. Approximately half of these deaths were randomly assigned for coding by physicians, for which VA structured questions and narratives were collected, and the remainder of the deaths were assigned to automated methods for coding using VA questionnaires with structured questions only. A randomly selected subset of the narratives from this RCT were translated into English, and are used in this study.

In the MDS and RCT datasets, each record is assigned a WHO International Classification of Diseases (ICD) version 10 code [27] by two specially trained physicians who independently and anonymously review each record. When the two assigned codes do not match (about 30% of records), the records undergo anonymous reconciliation, and persisting disagreements (about 15%) are adjudicated by a third senior physician. This process is standard for physician-coded VAs [20] and was conducted independently of developing our automated method.

**Fig. 1:**
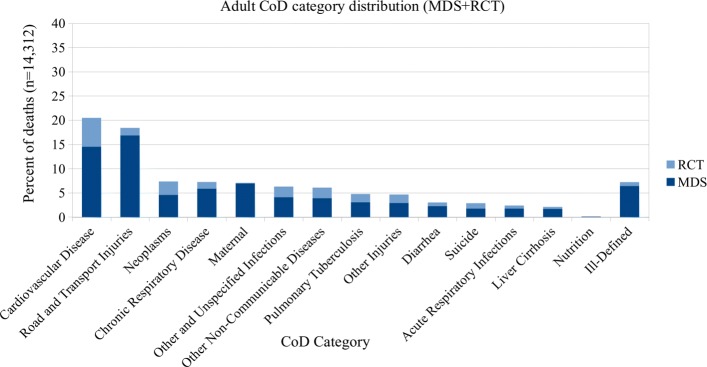
Adult CoD category distribution (15 categories)

**Fig. 2:**
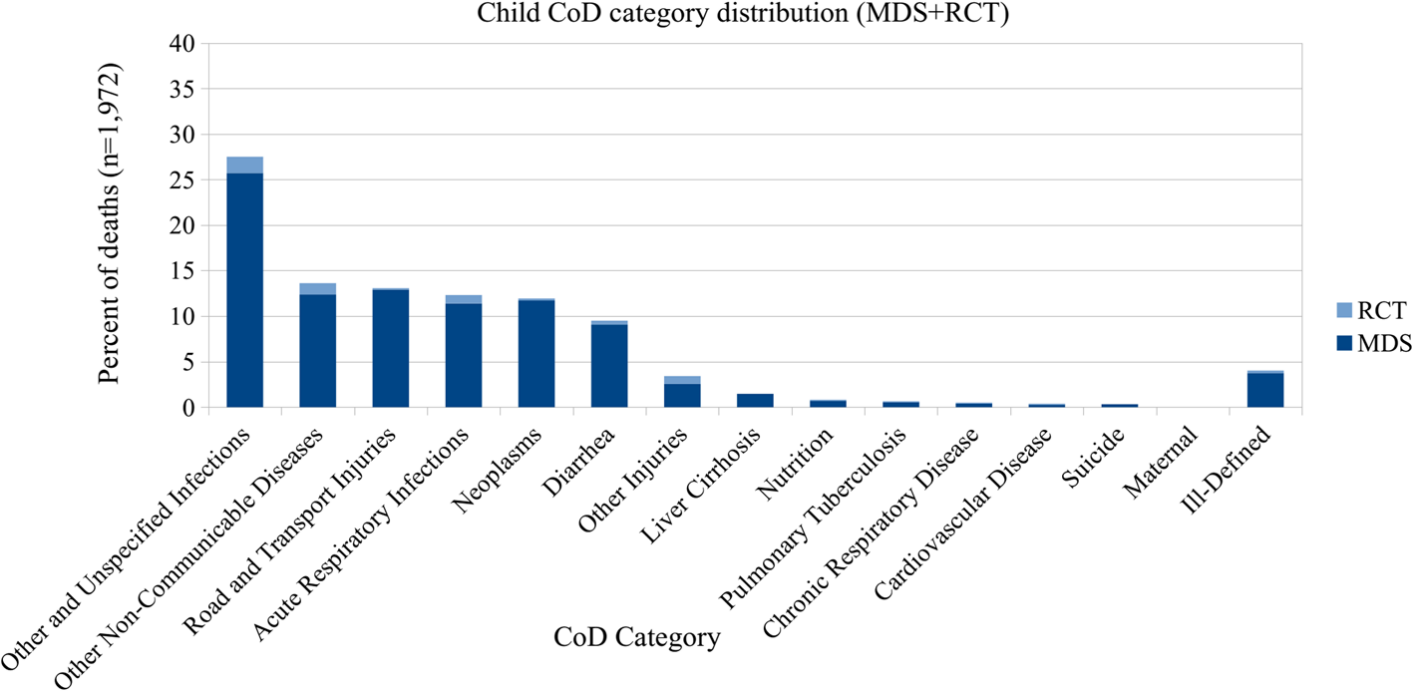
Child CoD category distribution (15 categories)

**Fig. 3:**
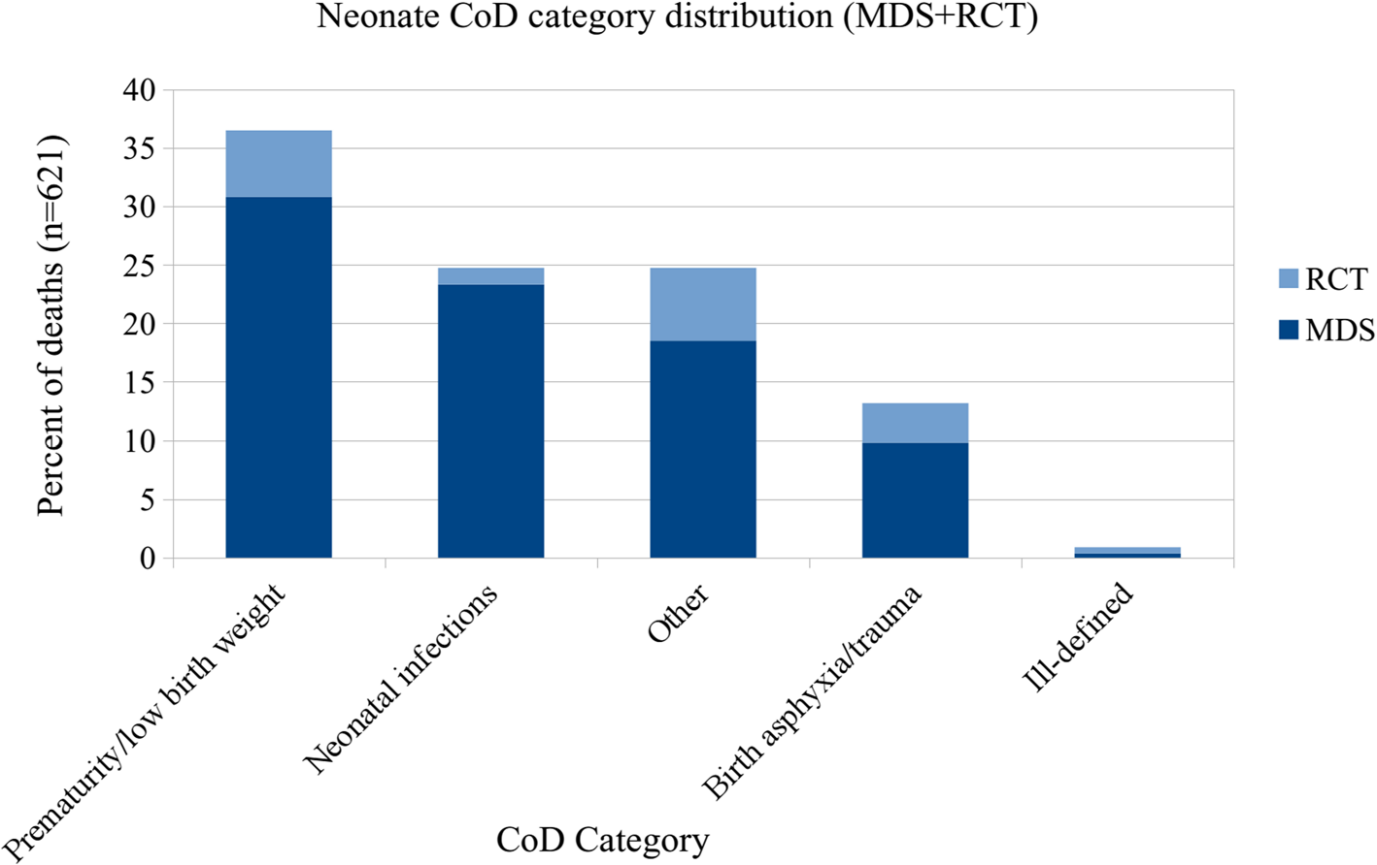
Neonate CoD category distribution (5 categories)

In the combined datasets there are over 500 individual ICD-10 codes. As the number of CoD categories used by other published VA studies ranges from 6 to 62 [14–18, 21], we use previously published CoD groupings [17] which are broader groupings of the WHO VA 2012 standardized CoD categories [22]. In this categorization scheme, the codes are grouped into 15 CoD categories for records of adult (15–69 years) and child (29 days–14 years) deaths, and 5 categories for records of neonatal (< 29 days) deaths. These groupings are based on an earlier evaluation [3] that best outlined the ability to use the maximal number of ICD-10 codes, which were all available for unrestricted use by the coding physicians in the MDS. See Tables 1 and 2 for CoD categories, and Additional file 1 for the complete mapping of ICD-10 codes to CoD categories. Figures 1, 2, and 3 show the distribution of CoD categories for each age group.

**Table 1:** CoD categories used for adult deaths (15–69 years), and child deaths (29 days–14 years)

Acute respiratory infections
Diarrhea
Pulmonary Tuberculosis
Other and unspecified infections
Neoplasms
Nutrition
Cardiovascular disease
Chronic respiratory disease
Liver cirrhosis
Other non-communicable diseases
Road and transport injuries
Other injuries
Ill-defined
Suicide
Maternal

**Table 2:** CoD categories used for neonatal deaths (<29 days)

Prematurity/low birth weight
Neonatal infections (not including tetanus)
Birth asphyxia/trauma
Ill-defined or cause unknown
Other (all other ICDs not included in above)

**Fig. 4:**
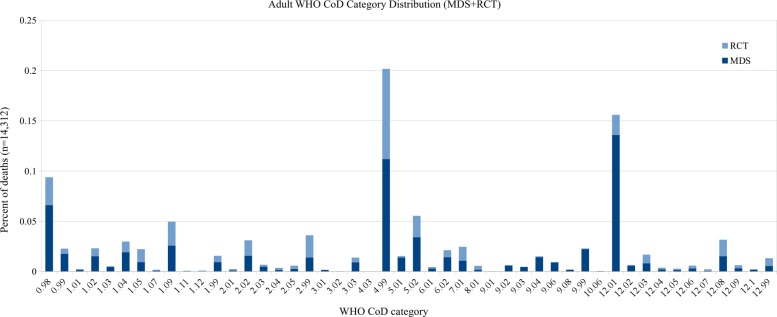
Adult CoD category distribution (48 WHO categories)

**Fig. 5:**
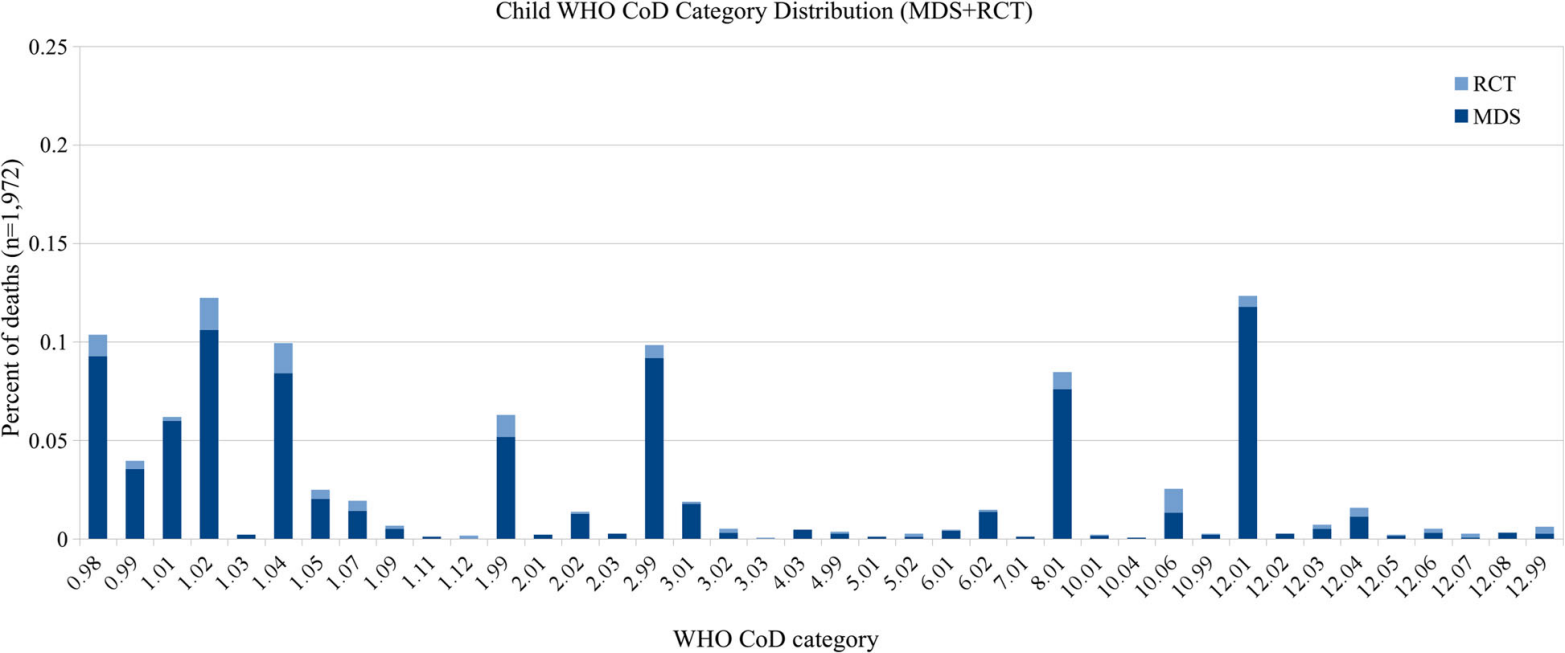
Child CoD category distribution (39 WHO categories)

**Fig. 6:**
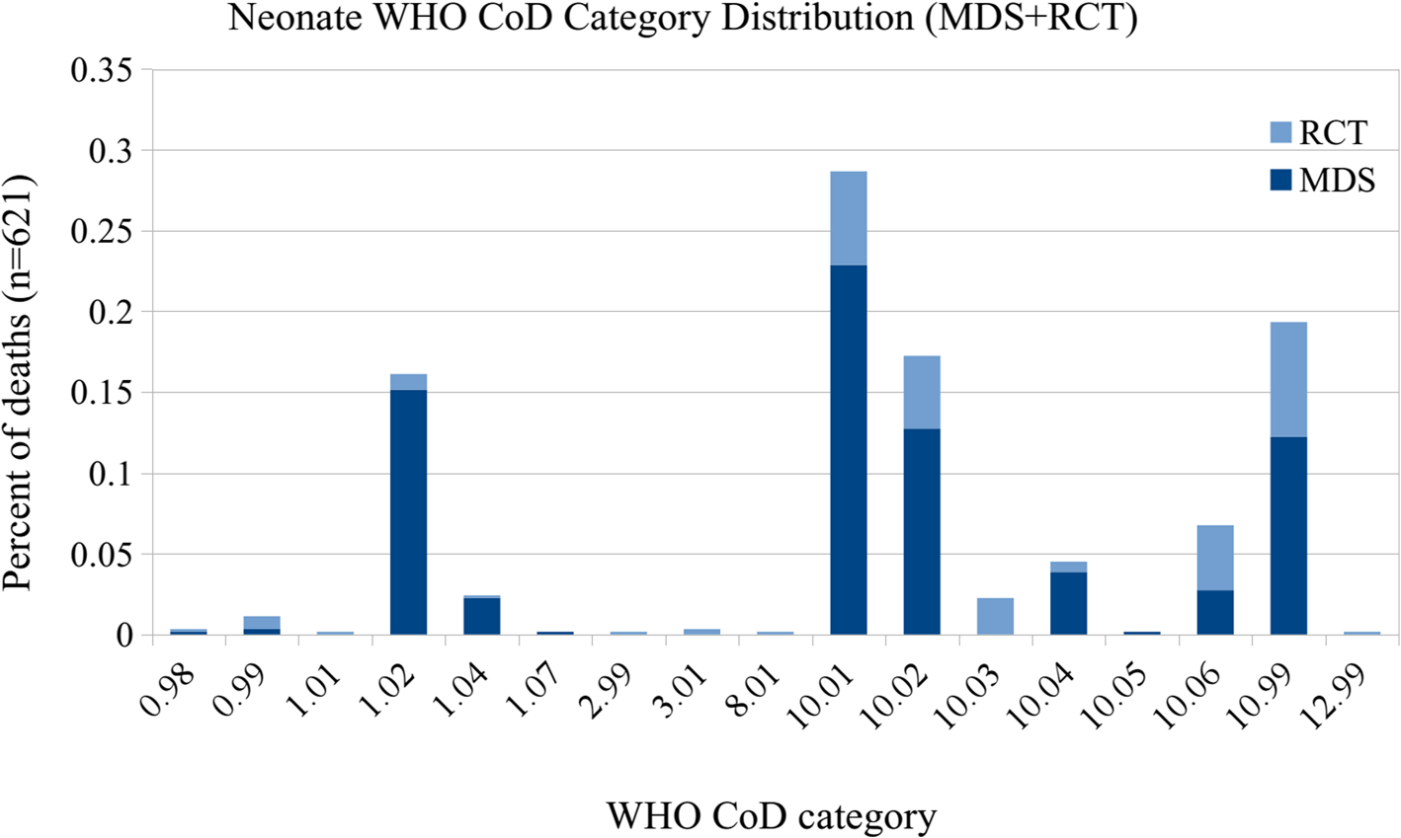
Neonate CoD category distribution (17 WHO categories)

For comparison, we also present results using the standard WHO 2016 categories, although we note that the distribution of these categories in the MDS data is very skewed, with many classes having only a few examples and a few classes having thousands of examples. Of the 62 possible categories, only 48 appear in the adult dataset, 39 in the child dataset, and 17 in the neonatal dataset. See Figs. 4, 5, and 6 for the distribution of the WHO categories in the dataset.

We also train and test our models on the Agincourt dataset, which is composed of coded VA records of community deaths in South Africa [28]. See Table 3 for details of the datasets.

**Table 3:** Description of datasets used. MDS: Million Death Study dataset, RCT: Randomized Control Trial dataset

	MDS	RCT	MDS+RCT	Agincourt
Adult records (15–69 years)	9,207	5,105	14,312	8,151
Child records (29 days–14 years)	1,717	255	1,972	1,674
Neonatal records (<29 days)	451	170	621	197
Region	India	India (Gujarat, Punjab)	India	South Africa

Since VA narratives are often handwritten and then transcribed and perhaps translated, there are frequent spelling errors and grammatical inconsistencies due to varying levels of experience of the surveyors and quality of the translations. In addition, medical symptoms are often described in non-medical or colloquial terms by the non-medical surveyors. Although the information is very often interpretable by medical professionals, the grammatical inconsistencies can make it difficult for automated systems to handle. In order to avoid some of these issues, we focus on individual words. See Table 4 for some examples of narrative text from the MDS dataset.

**Table 4:** Two example narratives (adult deaths)

Narrative	Physician certified CoD category
Heart failure. The patient death due to breathlessness. The person suffering paralysis and stroke lost on year with chest pain very pressure after then person was head.	Cardiovascular disease
One day 13/03/01 he fell ill with some fever and chest pain who called the Doctor. On 15/03/01 the deceased was crying in the chest pain and high fever. We were ready to shift. The patient to the Hospital, some water came out from the deceased mouth and closed his eyes and passed away.	Acute respiratory infections

### Implementation of metrics

In order to evaluate chance-corrected CSMF accuracy, we applied the Monte Carlo calculation described by Flaxman et al. [12] with 10,000 iterations, and found the mean CSMF accuracy of randomly assigning CoD categories to be.646 for the neonatal dataset (5 CoD categories),.641 for the child dataset (15 CoD categories), and.643 for the adult dataset (15 CoD categories). We use these values as the mean for chance-correcting CSMF accuracy because they are specific to our dataset, although they are very close to the value of.632 that Flaxman et al. reported.

Since the records for each test set and training set are selected randomly for each crossvalidation iteration, we expect the test distributions to be similar to the training distributions. Some VA studies have re-sampled their training and test set to create uniform distributions in order to avoid the model learning to assign CoD categories to individual records based on the frequency of the CoD categories [17, 23]. However, we chose not to do so because some CoD categories have a very small number of records and achieving a reasonably sized test set would require us to oversample some categories extensively, which would not constitute a rigorous evaluation of our method.

### Machine learning models for text classification

Like the MDS, the RCT data was also collected in India and follows a similar protocol to the MDS [3, 25, 26], so the two sets were combined to create a larger dataset with which to train and test our method. Unlike these datasets, the Agincourt data was captured in South Africa and has greater variations in protocol [28], and hence was not combined with the other datasets. Early experiments showed that the model performed better with more training data, which is typical of machine learning classifiers. The datasets were preprocessed as follows. Spelling was corrected by using the PyEnchant Python library [29] with an English dictionary and a short hand-crafted dictionary containing common terms that appear in the narratives. The text was subsequently lowercased and punctuation separated from words. A set of 160 stopwords (such as *and*, *because*, *for*) were removed from the narratives^1^. The remaining words were stemmed (i.e. morphological endings removed)^2^; for example, the stem of *crying* is *cry*.

The features that we use for CoD classification are word frequency counts from the narrative and one feature that indicates whether the record is of an adult, child, or neonatal death. We compute the ANOVA F-value^3^ for each feature, which calculates the ratio of the variance between the means of the feature values for each of the CoD categories, to the variance within each class. If the means are significantly different between CoD categories and the variance within categories is small, then the feature is likely to be discriminative. We keep only the features with the highest F-values, reducing the space from over 4000 to several hundred features, depending on the model (the actual number is chosen by hyper-optimization).

For our classifiers, all models except the neural network are created in Python with scikit-learn [30]. Each classifier is optimized^4^ for 100 runs for model parameters and the number of features, using a small subset of the MDS data. The models are optimized separately so we are comparing the best version of each model. The naïve Bayes classifier, which assigns a CoD category to a record using the independent conditional probabilities for each feature, uses the best 200 features (as chosen by ANOVA). The random forest model, which uses a combination of learned decision trees to classify new data points, uses the best 414 features and 26 trees.

Support vector machines (SVMs) are commonly used models that learn to classify data by maximizing the margin between categories in the training data, using a kernel function that maps the input features to higher dimensional space. Our SVM model is an aggregate of one-vs-rest SVMs with linear kernel functions, using 378 features.

Neural networks are made up of layers of simulated neurons with connections between the layers that can transmit information. The neural network model we use is a feed-forward network with one hidden layer (297 nodes, chosen by optimization) created with Keras [31], using Theano [32] as the backend. It uses 398 features and rectified linear units (ReLUs) as the activation function (the function that computes the output of an artificial neuron in the network given input values and learned weights).

For the adult and child datasets, each training set is augmented with all the data from the other two datasets. In general, we found that the classifiers perform better with extra training data, especially for the smaller child dataset. For neonatal records, the models are trained only with neonatal data because these records use a different set of CoD categories.

## Results

**Fig. 7:**
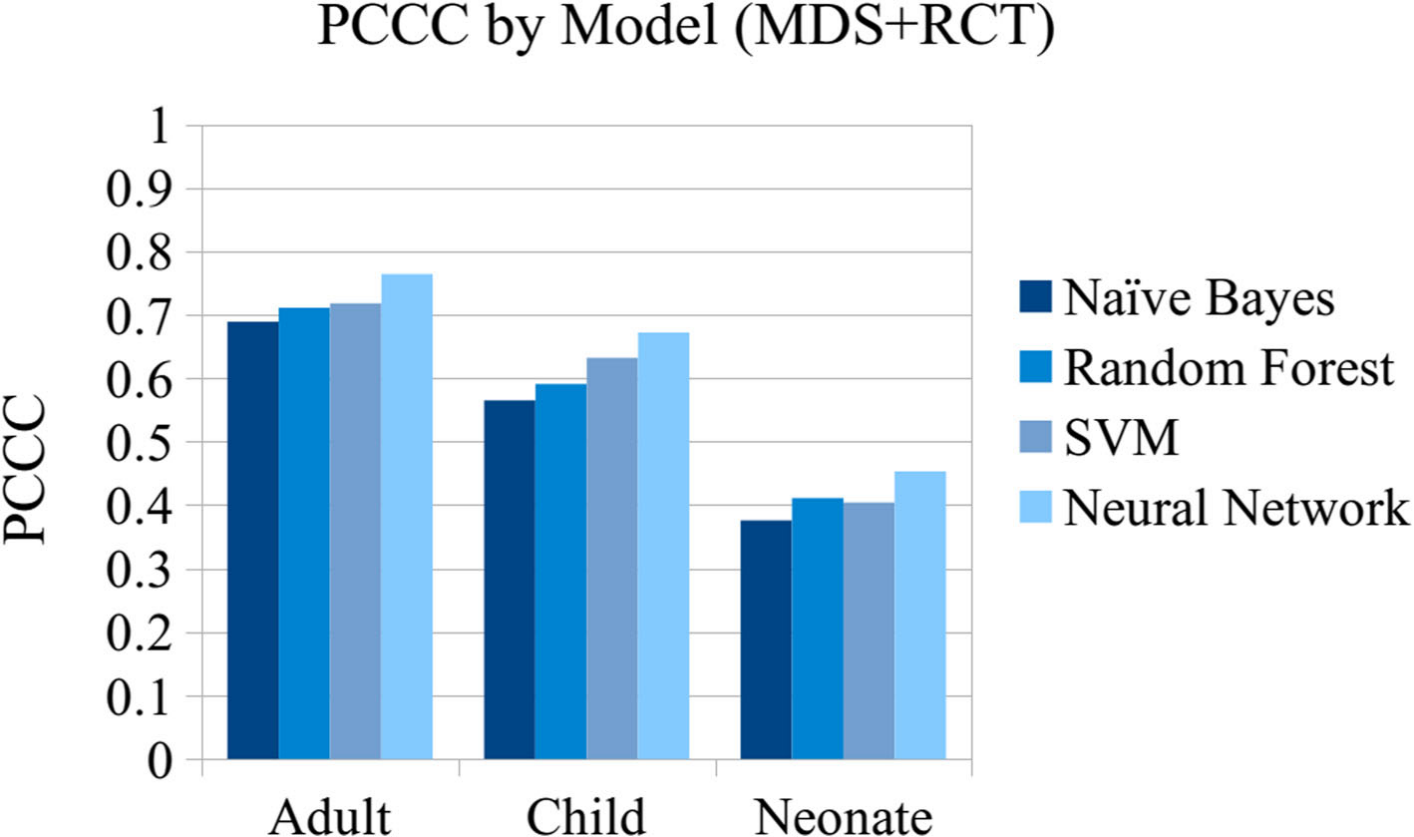
Individual level results comparison (MDS+RCT)

**Fig. 8:**
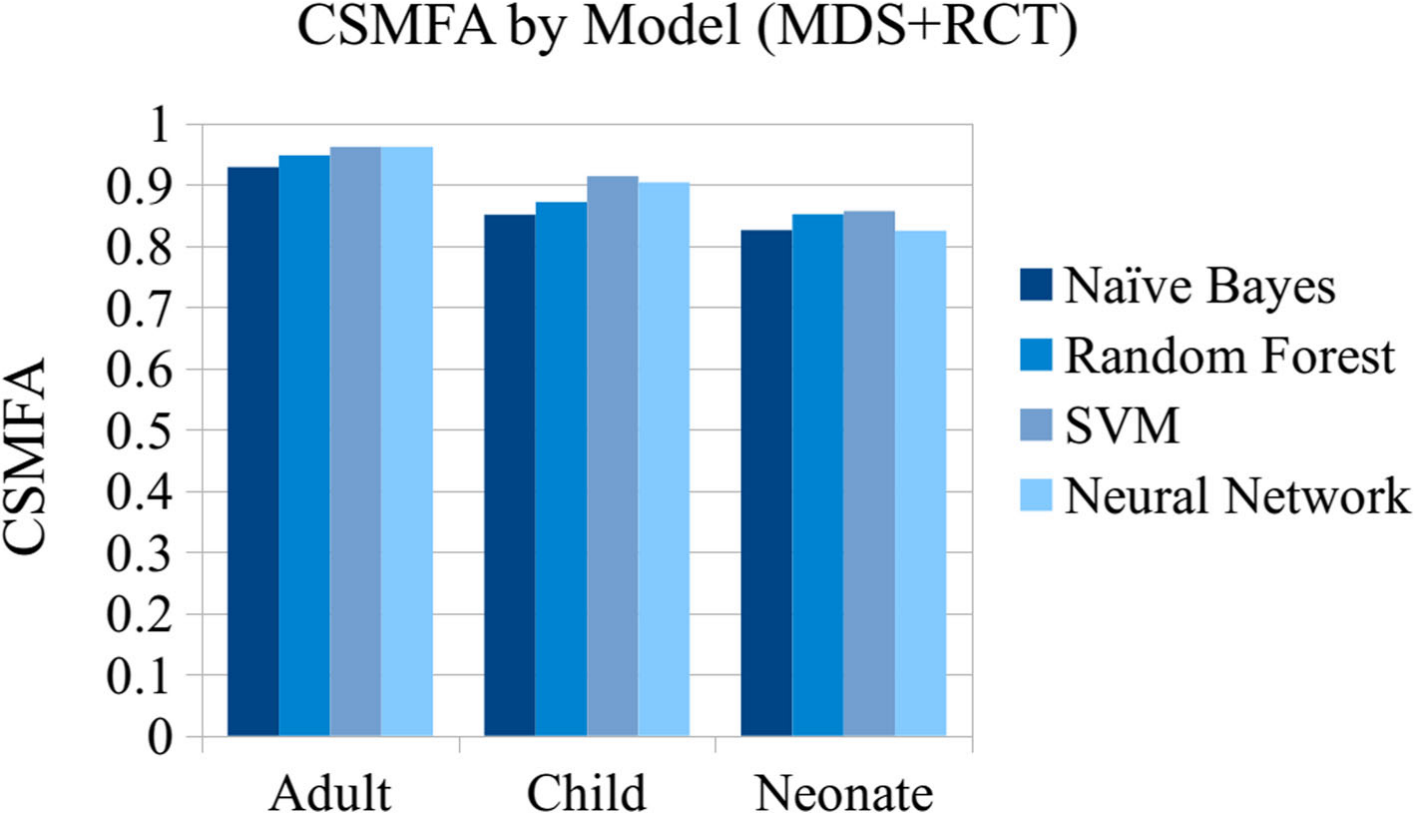
Population level results comparison (MDS+RCT)

Table 5 shows the mean scores for each classifier using 10-fold cross-validation with the combined MDS and RCT data. Each of the 10 test splits contained approximately 1,204 adult records, 185 child records, and 57 neonatal records. Overall, the neural network performs the best in terms of sensitivity, with.770 for adults,.695 for child records, and.576 for neonatal records. However, for CSMF accuracy the best performance is achieved by the SVM and neural network classifiers on adult records (.962), and the SVM on child records (.914) and neonatal records (.857). See Figs. 7 and 8 for a comparison of the PCCC and CSMF accuracy scores of the four machine learning models.

**Table 5:** Mean scores on the combined MDS and RCT datasets for each of the four classifiers

	Precision	Sensitivity	F _1_	PCCC	CSMFA	CCCSMFA
Adult (15–69 years)						
Naïve Bayes	.710	.710	.704	.689	.929	.801
Random forest	.733	.730	.728	.711	.948	.854
SVM	.746	.737	.740	.718	**.962**	**.894**
Neural network	**.773**	**.770**	**.770**	**.764**	**.962**	**.894**
Child (29 days–14 years)						
Naïve Bayes	.647	.595	.608	.565	.851	.585
Random forest	.687	.620	.638	.591	.872	.643
SVM	.686	.658	.666	.632	**.914**	**.760**
Neural network	**.719**	**.695**	**.698**	**.672**	.904	.733
Neonate (<29 days)						
Naïve Bayes	.507	.516	.493	.376	.826	.509
Random forest	.534	.542	.524	.411	.852	.581
SVM	.537	.538	.524	.404	**.857**	**.597**
Neural network	**.579**	**.576**	**.556**	**.453**	.825	.507

Adult and child results classified into 15 categories; neonatal records into 5 categories. Bold indicates the best score in each column for each age group. PCCC: partially chance-corrected concordance, CSMFA: cause-specific mortality fraction (CSMF) accuracy, CCCSMFA: chance-corrected CSMFA

In comparison to our model’s sensitivity of.770 for adult deaths and.695 for child deaths, Miasnikof et al. [17] reported a mean sensitivity of.57 on MDS checklist data from child and adult deaths with their naïve Bayes classifier and 16 CoD categories. They compared their results to InterVA-4 on the Million Death Study data, which achieved.43, and the Tariff Method, which achieved.50 sensitivity. InSilicoVA reported a sensitivity of.341 using 34 CoD categories for adult deaths from the PHMRC dataset [24]. Danso et al. [19] reported a sensitivity of.406 with their SVM classifier using narrative features from a dataset of 6407 neonatal records and 16 CoD categories, and.616 using narrative and structured data features, while our model achieved.576 sensitivity for records of neonatal deaths using only the narrative.

In comparison to our neural network classifier’s CSMF accuracy scores of.962 for adult deaths and.914 for child deaths, the King-Lu method achieved.96 on MDS data [7] (although the King-Lu method does not assign CoD categories to individual records), Miasnikof reported a CSMF accuracy of.88 for their model,.71 for InterVA-4,.57 for the Tariff Method, and InsilicoVA reported.85 CSMF accuracy.

Using the WHO categories, the SVM model performs the best for individual classification, as seen in Table 6 (.654 PCCC for adult records,.512 for child records, and.431 for neonatal records). The larger number of WHO CoD categories (48 for adult records, 39 for child records, and 17 for neonatal records) may account for the lower scores across all models due to limited training data for each CoD category. The poor performance of the neural network on the neonatal datasets is likely the result of the limited number of records available for training, as neural networks typically require a large amount of data to learn good parameters.

**Table 6:** Mean scores using WHO categories on the combined MDS and RCT datasets for each of the four classifiers

	Precision	Sensitivity	F_1_	PCCC	CSMFA	CCCSMFA
Adult (15–69 years)						
Naïve Bayes	.591	.593	.580	.583	.869	.643
Random forest	.644	.647	.634	.638	.905	.742
SVM	**.665**	**.662**	**.655**	**.654**	**.908**	**.751**
Neural network	.630	.654	.620	.646	.840	.567
Child (29 days–14 years)						
Naïve Bayes	.493	.402	.427	.379	.768	.369
Random forest	**.570**	.507	.514	.488	**.807**	**.476**
SVM	.567	**.530**	**.528**	**.512**	.796	.446
Neural network	.512	.494	.474	.474	.753	.330
Neonate (<29 days)						
Naïve Bayes	.434	.469	.435	.399	.797	.448
Random forest	.424	.455	.426	.384	.798	.450
SVM	**.505**	**.497**	**.476**	**.431**	**.813**	**.492**
Neural network	.328	.361	.306	.278	.634	.007

Adult: 48 categories, child: 39 categories, neonate: 17 categories. Bold indicates the best score in each column for each age group. PCCC: partially chance-corrected concordance, CSMFA: cause-specific mortality fraction (CSMF) accuracy, CCCSMFA: chance-corrected CSMFA

See Table 7 for results on the Agincourt dataset. As with the MDS dataset, the neural network performs the best for adult records, with a sensitivity of.578 and PCCC of.547. For the Agincourt neonatal records, the naïve Bayes model performs the best (.526 sensitivity and.404 PCCC), likely because the dataset is so small. By comparison, Miasnikof et al. [17] reported an overall sensitivity of.48 and PCCC of.43 on the Agincourt dataset, and Desai et al. reported a PCC of.38 using the open source Tariff method and.39 using InterVA-4. Table 8 shows the results using the WHO categories, which are generally lower, as with the MDS+RCT dataset.

**Table 7:** Mean scores on the Agincourt dataset

	Precision	Sensitivity	F _1_	PCCC	CSMFA	CCCSMFA
Adult (15–69 years)						
Naïve Bayes	.517	.517	.513	.481	**.932**	**.814**
Random forest	.511	.517	.496	.480	.844	.577
SVM	.569	.566	.561	.543	.901	.730
Neural network	**.575**	**.578**	**.570**	**.547**	.918	.777
Child (29 days–14 years)						
Naïve Bayes	.488	.440	.435	.395	.761	.351
Random forest	.521	.502	.487	.463	.816	.501
SVM	.535	.518	.512	.479	**.872**	**.653**
Neural network	**.572**	**.562**	**.552**	**.527**	.869	.645
Neonate (<29 days)						
Naïve Bayes	**.532**	**.526**	**.483**	**.404**	.702	.191
Random forest	.409	.496	.427	.366	**.710**	**.213**
SVM	.387	.417	.371	.266	.693	.165
Neural network	.356	.412	.354	.259	.636	.012

CCCSMFA was calculated using.632 as the mean of random allocation, as suggested in [12]

**Table 8:** Mean scores on the Agincourt dataset using the WHO categories

	Precision	Sensitivity	F _1_	PCCC	CSMFA	CCCSMFA
Adult (15–69 years)						
Naïve Bayes	.433	.448	.431	.432	**.876**	**.662**
Random forest	.438	.464	.436	.448	.832	.543
SVM	**.502**	**.505**	**.491**	.490	.857	.612
Neural network	.470	.495	.451	.480	.750	.322
Child (29 days–14 years)						
Naïve Bayes	.378	.388	.370	.360	.793	.437
Random forest	.456	.450	.431	.425	.799	.453
SVM	**.471**	**.465**	**.452**	**.440**	**.816**	**.499**
Neural network	.388	.428	.374	.402	.667	.095
Neonate (<29 days)						
Naïve Bayes	.276	.384	.305	.296	.610	-.060
Random forest	.292	.369	.314	.279	.673	.111
SVM	**.391**	**.405**	**.373**	**.320**	**.733**	**.274**
Neural network	.156	.265	.179	.160	.502	-.353

CCCSMFA was calculated using.632 as the mean of random allocation, as suggested in [12]

## Discussion

Some have suggested that it might be better to replace the free-text portions with more detailed checklist items to avoid the overhead of manually collecting, transcribing, translating, and processing the narrative [24]. While structured data can be very useful, it is more time-consuming to collect, and currently does not capture information such as chronology and health-seeking behaviors that is often made available via the narrative. We have demonstrated that despite the varying quality of the narrative text, it can still be used to achieve high agreement with physician-determined CoD.

While most other methods achieve their results by using expert-driven features or a large amount of data from the structured questionnaire in addition to some narrative-based features (in the case of the Tariff method [23] and Danso et al. [18, 19]), our model uses only the narrative and thus can be trained and tested on any set of verbal autopsies that contain free-text narratives, and we are able to achieve comparable performance to previously reported automated methods using the MDS, RCT and Agincourt datasets.

The datasets we use are very similar, but not exactly the same as the ones used by Miasnikof et al. [17], because we only use the records that have transcribed narratives. Unfortunately, at the time of writing there was currently no freely available dataset with narratives that would facilitate a direct comparison on the individual level. However, these records are taken from the same populations and therefore we expect the distribution of causes to be similar.

A possible explanation for why our narrative-based classifiers performed better than that of Danso et al. [18], besides the differences in the dataset, is that not only did we train on more data, but we also performed feature selection and parameter optimization for each classifier, while Danso et al. only performed feature reduction for the SVM, and used the default parameters for all models. Our feature selection based on the correlation between features and categories helps to prevent overfitting to the training data and reduce computation time for our models. Some of the highest ranked features that were selected by the ANOVA module are words like *yellow*, *abdomen*, *weak*, *fever*, *cough*, etc, which clearly describe symptoms. Some of the features seemed to describe conditions or situations, such as *pregnancy*, *cancer*, and *tuberculosis*, and some were less obvious, such as *help*, *gradually*, and *one*.

Certain CoD categories have fewer misclassifications, most notably “Suicide" and “Road and transport injuries". Those narratives tend to be less complex since the CoD is well identified within the text. The most commonly confounded CoD categories were “Other non-communicable diseases" and “Ill-defined". The classifiers seem to have more trouble distinguishing between CoD categories that have a large variation in symptom patterns, which are also more difficult for humans to diagnose.

Given that physicians do not agree with each other 100% of the time on CoD, we cannot expect automatic classifiers to achieve perfect agreement with humans. Because the model reports its own level of confidence for each record, we can use these confidence scores to decide which codes to send for physician review and which to accept without review.

One disadvantage of our method is that some narratives are long and include background information that is not ultimately relevant to the CoD, such as a history of smoking or asthma when the subject died in a car accident. Sometimes the respondents mention what they believe to be the CoD in the narrative, which might or might not be the CoD that is subsequently determined by the physicians. The presence of these elements in the narrative could potentially cause a misclassification. While we may be willing to accept the same kinds of errors from the system that a physician might make, the system should not make simple mistakes that a human wouldn’t, such as ignoring more recent events (hit by car) and instead focusing only on earlier events from the person’s medical history (asthma).

This kind of error can arise because the word frequency counts do not take word order into account, and consequently, higher-level linguistic information such as negation and chronology is not captured. We plan to handle some of these issues in the future by using models that capture the sequence of the words, and we also plan to use temporal relation extraction to account for chronology. However, the present work provides a strong baseline for narrative-based automated VA coding.

## Conclusions

We have shown that a variety of narrative-based machine learning classifiers can be used for automated VA coding. This was previously demonstrated by Danso et al., but we extend this work to include neural network models and datasets from India and South Africa. Unlike most other methods, ours does not rely on a specific structured data format or questionnaire; it can be applied to any English VA narrative, and is more adaptable to different datasets and populations than methods that rely on structured data.

No current method for automatically determining CoD for VA records has sufficient accuracy to be a replacement for human doctors. However, we have shown that for adult deaths, the largest group of deaths in our dataset, that our method can achieve.770 sensitivity and over.90 agreement (CSMF accuracy) at the population level with physician-assigned CoDs. This demonstrates that narrative-based machine learning methods are a promising option for automated CoD coding of VA records. A large repository of openly available VA data with full narratives and physician-assigned cause of death would help in further development of such computational methods. Similar methods of text-based machine learning could be applied to other tasks in the healthcare domain, such as automatic diagnosis or treatment recommendations based on hospital records.

To improve our VA classification method, we are currently considering combinations of features from the structured data and the narrative in order to produce an automated CoD coding tool that is robust and reliable enough to be used in the field. In our ongoing work, we are using more linguistically motivated features that take into account context, chronology, and semantics, and we are also exploring alternative neural network architectures.

## Additional file

Additional file 1 Cause of death categories with corresponding ICD-10 codes (PDF 59 kb)

## Data Availability

The MDS and RCT datasets are the property of the Government of India and cannot be shared.

## Abbreviations

ANOVA: analysis of variance
CCC: Chance-corrected concordance
CCCSMFA: Chance-corrected CSMF accuracy
CoD: Cause of death
CSMF: Cause-specific mortality fraction
ICD: International classification of diseases
MDS: Million death study
PCCC: Partial chance-corrected concordance
PHMRC: Population health metrics research consortium
RCT: Randomized controlled trial
SVM: Support vector machine
VA: verbal autopsy
WHO: World Health Organization

## Footnotes

1 Danso et al. [18] also lowercased the text in their dataset but removed punctuation and did not remove stopwords or perform spelling correction.

2 We use the implementation of the Porter Stemmer provided in NLTK [33].

3 We use scikit-learn’s SelectKBest module with the f_classif function [30].

4 For optimization we use the hyperopt Python library[34].
