# Supplementary material for: Automatically determining cause of death from verbal autopsy narratives
Source: BMC Med Inform Decis Mak. 2019 Jul 9;19:127. doi: 10.1186/s12911-019-0841-9 (PMC6617656; doi:10.1186/s12911-019-0841-9)
Supplement: Supplementary file 1 — Cause of death categories with corresponding ICD-10 codes (PDF 59 kb) [file 12911_2019_841_MOESM1_ESM.pdf]

Additional file 1: Cause of death categories with corresponding ICD-10 codes

Cause of death	ICD-10 Range
**Adult (15 - 69 years) & child (29 days - 14 years)**	
Pulmonary tuberculosis	A15-A16, B90, J65
Diarrhea	A00-A09
Acute respiratory infections	H65-H68, H70-H71, J00-J22, J32, J36, J85-J86, P23, U04
Other and unspecified infections	A17-A33, A35-A99, B00-B17, B19-B89, B91-B99, C46, D64, D84, G00-G09, H10, H60, I30, I32-I33, K02, K04-K05, K61, K65, K67, K81, L00-L04, L08, M00-M01, M60, M86, N10, N30, N34, N41, N49, N61, N70-N74, P35-P39, R50, R75
Maternal conditions	A34, F53, O00-O08, O10-O16, O20-O99
Nutritional deficiencies	D50-D53, E00-E02, E40-E46, E50-E64, X53-X54
Neoplasms	C00-C26, C30-C45, C47-C58, C60-C97, D00-D48, D91, N60, N62-N64, N87, R59
Cardiovascular disease	E10-E14, G43, G45-G46, G81-G83, I00-I15, I20-I28, I31, I34-I52, I58-I84, I86-I99, R00-R01, R03, R55
Chronic respiratory diseases	J30-J31, J33-J35, J37-J64, J66-J84, J90-J99, R04, R06, R84, R91
Liver cirrhosis	K70-K77
Other noncommunicable diseases	B18, D55-D63, D65-D83, D86, D89, E03-E07, E15-E35, E65-E90, F00-F52, F54-F99, G10-G37, G40-G41, G50-G80, G84-G99, H00-H06, H11-H59, H61-H62, H69, H72-H95, I85, K00-K01, K03, K06-K31, K35-K38, K40-K60, K62-K64, K66, K78-K80, K82-K93, L05, L10-L99, M02-M54, M61-M85, M87-M99, N00-N08, N11-N29, N31-N33, N35-N40, N42-N48, N50-N59, N75-N86, N88-N99, Q00-Q99, R05, R10-R23, R26-R49, R56, R63, R70-R74, R76-R77, R80-R82, R85-R87, R90
Road and transport injuries	V01-V99, Y85
Suicide	X60-X84
Other injuries	S00-S99, T00-T99, W00-W99, X00-X52, X55-X59, X85-X99, Y00-Y84, Y86-Y98
Ill-defined	R02, R07-R09, R25, R51-R54, R57-R58, R60-R62, R64-R69, R78-R79, R83, R89, R92-R94, R96-R99
**Neonate (0 - 28 days)**	
Neonatal infections (not including tetanus)	A20-A28, A32, A37-A44, A46, A48-A49, A68-A70, A74-A75, A77-A79, A81-A89, B95-B96, G00-G09, H10, H60, H65-H68, H70-H71, I30, I32-I33, I39-I41, J00-J22, J32, J36, J85-J86, K65, K67, K81, L00-L04, L08, M00-M01, M60, M86, N10, N30, N34, N41, N49, N61, O85, P23, P35-P39, P58-P59, U04
Birth asphyxia/trauma	P00, P02-P03, P10-P15, P20-P21, P24, P29, P50-P51, P90-P91, R06, W79, Z37
Prematurity/ low birthweight	O60, P01, P05, P07, P22, P25-P28, P52, P61, P77, P80, P92, R04
Other (all other ICDs not included in above)	A00-A09, A15-A19, A30-A31, A33-A36, A50-A67, A71, A80, A90-A99, B00-B09, B15-B27, B30, B33-B60, B64-B83, B85-B92, B94, B97, B99, C00-C97, D00-D48, D50-D53, D55-D89, E00-E02, E03-E35, E40-E46, E50-E56, E59-E61, E63-E64, E65-E90, F00-F99, G10-G99, H00-H06, H11-H59, H61-H62, H69, H72-H95, I00-I28, I31, I34-I38, I42-I99, J30-J31, J33-J35, J37-J47, J60, J64, J65, J66-J70, J80-J82, J84, J90-J99, K00-K03, K04-K05, K06-K60, K61, K62-K63, K70-K80, K82-K93, L05, L10-L99, M02-M54, M61-M85, M87-M99, N00-N08, N11-N29, N31-N33, N35-N40, N42-N48, N50-N51, N60, N62-N64, N70-N74, N75-N99, P04, P08, P53-P57, P60, P70-P72, P74-P76, P78, P81-P83, P93-P94, P96, Q00-Q99, R00-R01, R03, R05, R11-R23, R26-R27, R29-R36, R39-R49, R50, R55-R56, R59, R63, R70-R74, R75, R76-R77, R80-R82, R84-R87, R90-R91, S00-S99, T00-T98, U00, V01-V99, W00-W78, W80-W99, X00-X52, X53-X54, X57-X99, Y00-Y91, Y95, Y97-Y98
Ill-defined	R02, R07, R09-R10, R25, R51-R54, R57-R58, R60-R62, R64, R68-R69, R78-R79, R83, R89, R92-R99
